# Effects of dietary vitamin E deficiency on systematic pathological changes and oxidative stress in fish

**DOI:** 10.18632/oncotarget.13729

**Published:** 2016-11-30

**Authors:** Kaiyu Wang, Erlong Wang, Zhenyang Qin, Zhen Zhou, Yi Geng, Defang Chen

**Affiliations:** ^1^ Department of Basic Veterinary, Sichuan Agricultural University, Chengdu, Sichuan, China; ^2^ Key Laboratory of Animal Disease and Human Health of Sichuan Province, Sichuan Agricultural University, Chengdu, Sichuan, China; ^3^ Department of Aquaculture, Sichuan Agricultural University, Chengdu, Sichuan, China

**Keywords:** fish, vitamin E, deficiency, histopathology, ultrastructural pathology, Pathology Section

## Abstract

The aim of this study was to investigate the effects of dietary vitamin E deficiency on systematic pathological changes and oxidative stress in fish. A total of 320 healthy common carp (*Cyprinus carpio*) were randomized into four groups; the control group was fed a basal diet supplemented with 100 IUkg^-1^ of vitamin E, while the three experimental groups were fed the same basal diet with reduced vitamin E content (0, 25, or 50 IUkg^-1^). Findings showed that fish in the experimental groups mainly presented with sekoke disease, exophthalmia, leprnorthsis, and ascites. Histopathological and ultrastructural changes comprised nutritional myopathy with muscle fiber denaturation and necrosis, and multi-tissue organ swelling, degeneration, and necrosis. Compared with the control group, RBC count, hemoglobin content, vitamin E concentration, and superoxide dismutase activity were significantly lower in all three experimental groups. However, malondialdehyde content was considerably higher in experimental groups than in the control group. However, there was no difference in glutathione peroxidase activity among groups. In conclusion, dietary vitamin E deficiency (<100 IUkg^-1^) can cause severe injury and, in particular, oxidative damage in common carp. The oxidative damage might be a main influence caused by vitamin E deficiency in fish. These findings reveal the complete systematic pathological effect of vitamin E deficiency in common carp, which may be applicable to other fish and animals.

## INTRODUCTION

Vitamin E, discovered by Evans in 1922, is a lipid-soluble vitamin which functions as an antioxidant; it protects biological membranes, lipoproteins, and lipid stores against oxidation [1, 2]. It particular, it protects unsaturated fatty acids against free radical-mediated oxidation. In addition, vitamin E is essential for maintaining flesh quality, normal resistance of red blood cells to hemolysis, and capillary and heart muscle permeability [3, 4]. It cannot be synthesized and fish are vulnerable to vitamin E deficiency based on dietary intake [5]. It was reported that vitamin E deficiency in fish may cause muscular dystrophy, exudative diathesis, anemia, impaired erythropoiesis, erythrocyte fragility, skin discoloration, and ceroid pigment deposition [6]. However, it has been demonstrated that diets supplemented with vitamin E can improve growth performance, enhance immunity, increase oxidative stability and shelf-life [7, 8], and restore impaired immunity [9]. With the development of modern intensive aquaculture, fish growth increasingly relies on feedstuff. However, due to lack of supplementation, fat oxidation, and feed ingredient mildew, feedstuff is deficient in vitamin E in practical production. This leads to hypovitaminosis E, presenting poor growth and a thin back, and can even lead to morality.

Although the effects of vitamin E deficiency has been researched in specific organs of different fish species [4, 5, 8, 10-12], the overall complete and systematic pathological effect has not been reported. The objectives of this study were to investigate the clinical symptoms, histological and ultrastructural pathological changes, and serum oxidative stress including Red Blood Cell (RBC) count, hemoglobin content, vitamin E concentration, superoxide dismutase (SOD) and glutathione peroxidase (GSH-Px) activities, and malondialdehyde (MDA) content under dietary vitamin E deficiency conditions in common carp (*Cyprinus carpio*).

## RESULTS

### Clinical symptoms and autopsy observations

During the entire experiment, morbidity and mortality in groups I, II, and III were 47.5% and 22.5%, 22.5% and 12.5%, and 10.0% and 5.0%, respectively, while both values in group IV (control) were 0 (Table 1). In addition, fish in groups I, II, and III presented with obvious clinical lesions, successively. Lesions appeared the earliest and were the most severe in group I, followed by group II, and appeared the latest and were less severe in group III, while fish in group IV were normal (Table 2).

**Table 1:** Morbidity and mortality of vitamin E deficiency in common carp

Groups	VE Dose (IUkg^-1^ )	Numbers	Clinical Sick / Dead Number	Time (weeks)				Morbidity (%)	Mortality (%)
				5	10	15	20		
I	0	40	Sick No.	0	5	10	19	47.5	22.5
			Dead No.	0	0	4	9		
II	25	40	Sick No.	0	0	4	9	22.5	12.5
			Dead No.	0	0	0	5		
III	50	40	Sick No.	0	0	2	4	10	5
			Dead No.	0	0	0	2		
IV (Control)	100	40	Sick No.	0	0	0	0	0	0
			Dead No.	0	0	0	0		

Notes: One or more of the sekoke disease, exophthalmia and leprnorthsis were considered as morbidity.

**Table 2:** Incidence of sekoke disease, exophthalmia, leprnorthsis, and rachiocamposis in vitamin E deficient fish

Group	Vitamin E dose (IUkg^−1^)	*n*	Sekoke disease (%)	Exophthalmia (%)	Leprnorthsis (%)	Rachiocamposis (%)
I	0	40	35.0	22.5	20.0	17.5
II	25	40	20.0	12.5	10	15
III	50	40	7.5	5	5	5
IV (Control)	100	40	0	0	0	0

**Figure 1:**
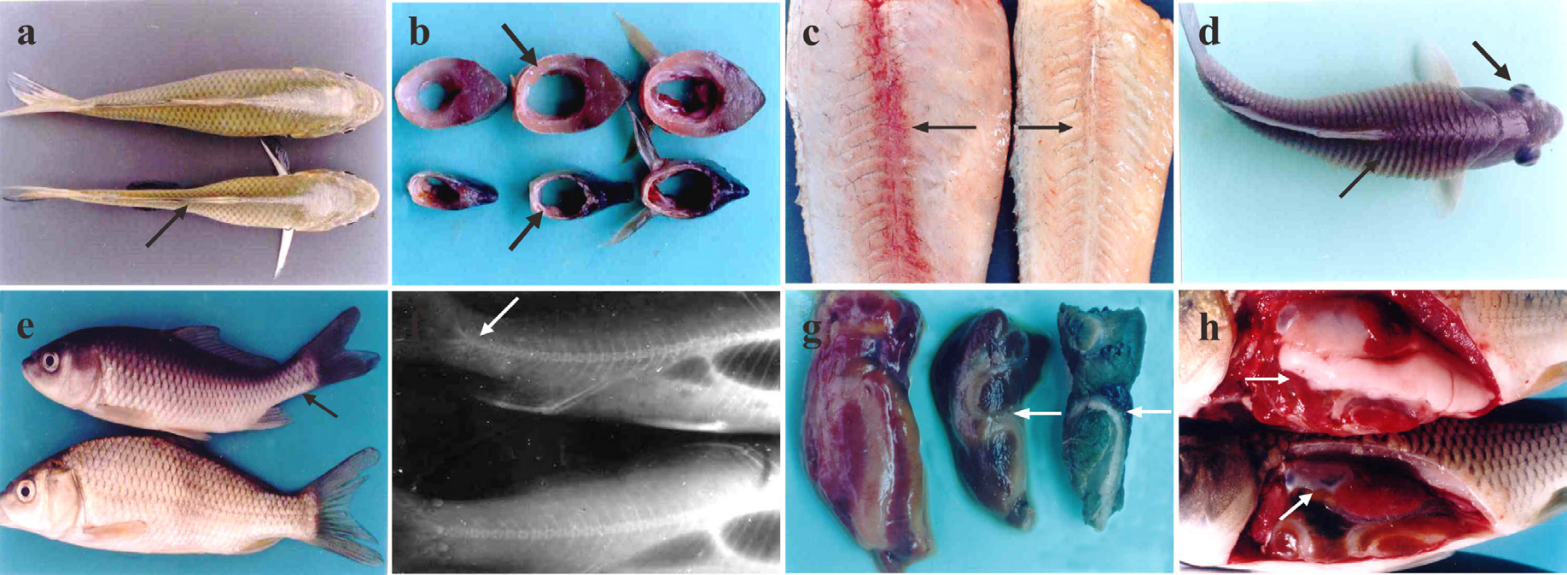
Clinical symptoms of vitamin E deficient fish **a**. Vitamin E deficient fish (below) with back muscle atrophy showing sekoke disease (↗); normal fish above. **b**. Muscle cross section in vitamin E deficient fish; muscle becomes thinner and atrophic (↗); the cross section of muscle in normal fish (↗). **c**. The red muscle color in vitamin E deficient fish fades, indicating white muscle disease (→), normal fish (←). **d**. Leprnorthsis (↗) and exophthalmia in the vitamin E deficient fish (↘). **e**. Tail of the sick fish is upturned (↖); normal fish below. **f**. X-radial photograph. Rachiocamposis in sick fish (↙), normal fish below. **g**. Green liver in vitamin E deficient fish (←). **h**. Gonads exhibiting atrophy (↗), normal fish above.

Throughout the experiment, common clinical signs in sick fish mainly included poor growth, inappetence, emaciation, sekoke disease, exophthalmia, leprnorthsis, ascites, and rachiocamposis. The predominant symptoms of fish in group I, including emaciation and inappetence, appeared the earliest at the 8^th^ week, and these fish became more severely emaciated with skeletal muscle atrophy after the 10^th^ week, leading to sekoke disease (Figure 1a). The back muscle thickness in sick fish was only 1/4 to 1/2 that of normal fish on the transverse section (Figure 1b). When removing the skin from sick fish, the color of the red muscle around the vertebral column had faded, which made it difficult to distinguish from the white muscle (Figure 1c).

After the 12^th^ week, the sick fish exhibited edema with upright squama and leprnorthsis (Figure 1d). Moreover, all of the sick fish with leprnorthsis showed exophthalmia on unilateral or bilateral sides (Figure 1d). By the 13^th^ week, some sick fish became malformed with an upturned tail (Figure 1e), and X-ray examination indicated that the caudal spinal column was markedly upturned (Figure 1f). In addition, the livers of sick fish became green, exhibiting “green liver diseases” (Figure 1g) with hepatomegaly; and the volume of liver was 1.5 times larger than normal. The kidneys were enlarged and hemorrhaged on the surface. Gonads (testis and ovaries) were stunted and only 1/3-1/2 the size of the control (Figure 1h). By the 15^th^ week, fish in groups II and III also showed similar symptoms successively, while fish in group IV were normal.

### Histopathological changes

Throughout the whole experiment, vitamin E deficiency caused dose- and time-dependent histopathological changes in main tissues and organs (including skeletal muscles, brain, gills, heart, liver, spleen, and kidneys) in fish from groups I, II, and III. The more severe the vitamin E deficiency, the more severe and earlier the pathological changes. For instance, fish in group I showed the earliest pathological changes, followed by groups II and III, while fish in group IV had no obvious histopathological changes. The histopathological changes in tissues and organs of vitamin E deficient fish were as follows:

#### Muscles

**Figure 2:**
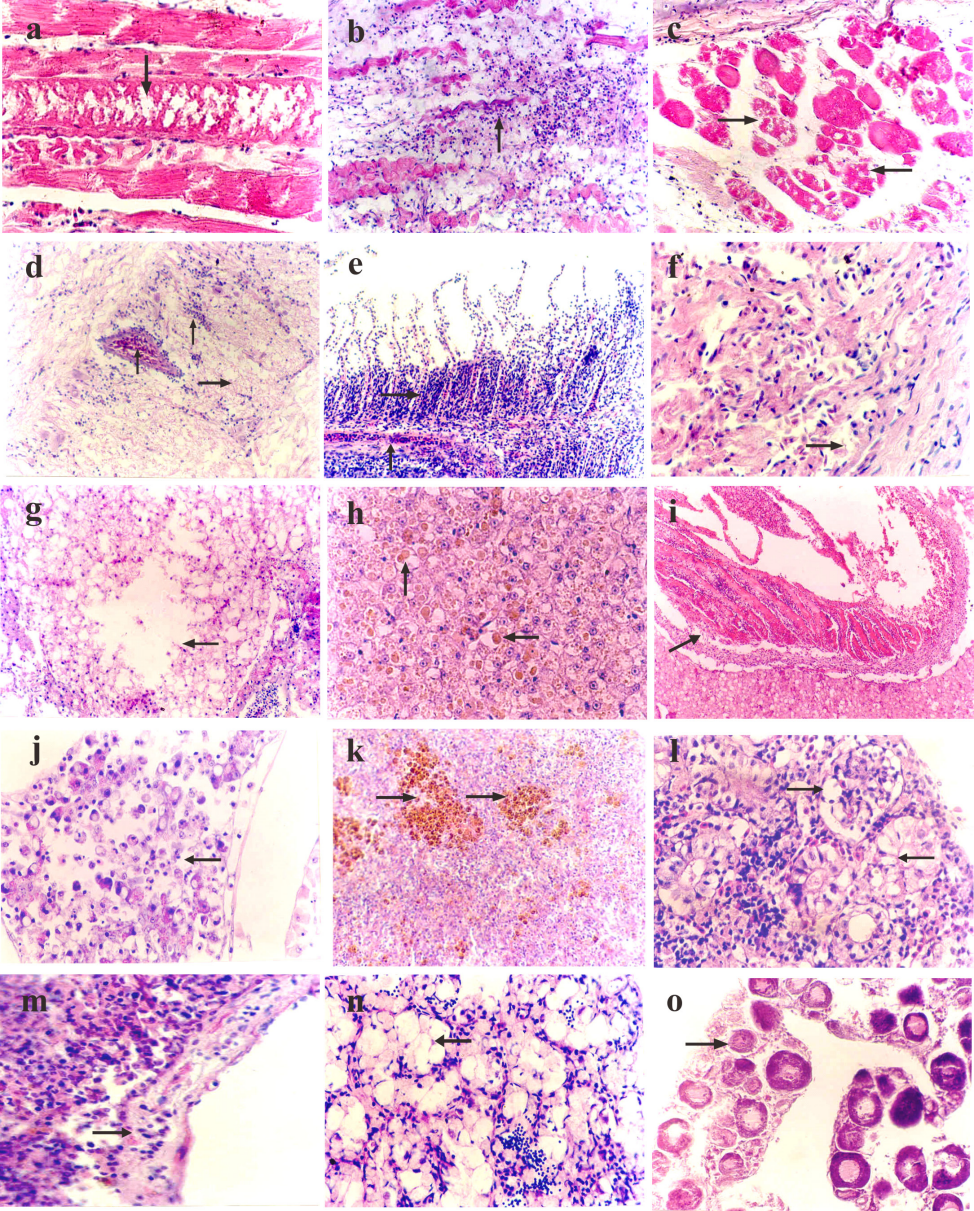
Histopathological changes in vitamin E deficient fish **a**. Skeletal muscle swelling and degeneration, with dissolving muscle plasm (↓), hematoxylin and eosin (H&E) × 400. **b**. Atrophy, degeneration, and necrosis of skeletal muscle, with the infiltration of inflammation cells (↑), H&E × 200. **c**. Musculus ocularis showing degeneration and necrosis (→), H&E × 400. **d**. Brain parenchyma loosens and exhibits edema (→), the capillaries show hyperemia, the vascular space widens with the infiltration of lymphoid cells (↑), H&E × 200. **e**. Hyperplasia of epithelia on the bottom of gill lamellae (→), and hyperemia of capillaries of gill lamellae (↑), H&E × 200. **f**. Cardiac muscle fiber degeneration, the cross striations are ambiguous or lacking (→), H&E × 400. **g**. The liver cells showing vacuolar degeneration and lysis (←), H&E × 200. **h**. Rounded or oval brown ceroid particles (↑ ←) present in liver cells, H&E × 400. **i**. Mixed thrombus (↗) in the liver interlobular vein, H&E × 200. **j**. Pancreatic cell degeneration and necrosis (←), H&E × 400. **k**. Extensive hemosiderosis observable in spleen tissues (↑), and the number and volume of melanin macrophages center is elevated, H&E × 200. **l**. Vacuolar degeneration in renal tubular epithelial cells (←), glomerulus swelling with cellular proliferation (→), H&E × 400. **m**. Vessel wall of head kidney loosening and incrassating with endothelial cell swelling and defluxion (→), H&E × 400. **n**. Sparse spermatocytes and spermatids in the seminiferous lobules (←), H&E × 200. **o**. Ovary presenting edema; the ovary cells develop slowly (→), H&E × 200.

The histopathological changes of skeletal muscles mainly presented muscular nutritional necrosis; the muscle fibers became swollen, with degeneration and necrosis, dissolved muscle plasm (Figure 2a), and lymphocyte and monocyte infiltration (Figure 2b).

#### Eyes

The choroid presented edema, loosened and thickened, along with hyperemia of the capillaries, and some lymphocyte and monocyte infiltration. The pathological changes of the musculus ocularis were similar to the skeletal muscles described above, mainly presenting as muscular nutritional necrosis with muscle fiber swelling, degeneration, and disintegration (Figure 2c), and the space between muscle fibers widening and edema.

#### Brain

The brain parenchyma of sick fish became loose and exhibited edema, there was hyperemia of the capillaries, the vascular space widened with infiltration of lymphoid cells (Figure 2d), nerve cells swelled, and denaturation with gliosis occurred, forming glial nodules.

#### Gill

Various degrees of epithelia hyperplasia were observed on the bottom of gill lamellae in sick fish, with a few mucous cells and granular cell hyperplasia. In addition, the capillaries of the gill lamellae exhibited hyperemia (Figure 2e). In time, the hyperplasia of the epithelia became more severe and obvious, with proliferous epithelia, and, in some severely affect areas, there was almost a permeation of mucous cells and granular cells into the whole gill lamellae space.

#### Heart

The epicardium loosened and thickened, and exhibited edema. Fish also presented with lymphocyte and monocyte infiltration, cardiac muscle fiber degeneration, ambiguous or missing cross striations, pale sarcoplasm, and even necrosis and dissolution with the widening of the muscle space (Figure 2f).

#### Liver

Hepatocyte swelling, an increase in volume, and granular degeneration were observed. In time, hepatocytes exhibited extensive vacuole degeneration and dissolvement, which resulted in the formation of dissolving stoves of different sizes (Figure 2g). Some of the severely affected liver cells were rounded or oval brown ceroid particles of different sizes (Figure 2h). Moreover, thrombosis was occasionally visible in interlobular veins (Figure 2i).

#### Pancreas

Swelling and volume increases were observed in the glandular cells. As the disease progressed, the glandular cells exhibited necrosis, the normal structure of the pancreas was disrupted, and islet cells presented denaturation and necrosis (Figure 2j).

#### Spleen

The splenic sinus presented hyperemia and hemorrhage, the lymphocytes decreased comparatively, and the reticulate endothelium cells swelled with hyperplasia. Extensive hemosiderosis was observed in spleen tissues, and the number and volume of melanin macrophages increased in the center (Figure 2k).

#### Kidney

The glomerulus was enlarged, and the vascular endothelial and reticular cells became swollen and exhibited hyperplasia. Renal tubular epithelial cell swelling and vacuolar degeneration, with numerous vacuoles of different sizes was also observed in cells (Figure 2l). The epithelia, with serious denaturalization, separated from the basement membrane and fell off into the lumen.

#### Head kidney

The blood sinus in the parenchyma appeared dilated with hyperemia. Lymphoid tissue decreased relatively, the vessel wall loosened and thickened, and endodermis cells swelled and fell off (Figure 2m).

#### Gonads

The testis of male sick fish showed reductions in the diameter of seminiferous lobules, and the quantity of spermatogonium, secondary spermatogonium, and spermatids in the seminiferous lobules. In some cases, spermatogonium, secondary spermatogonium and the spermatids inside were absent, with only edematous fluid present (Figure 2n). The ovaries of female sick fish exhibited edema and reduced quantity of oocytes with slow ovary cell development (Figure 2o).

### Ultrastructural observations

The ultrastructural changes in tissues and organs of vitamin E deficient fish were as follows:

#### Intestines

**Figure 3:**
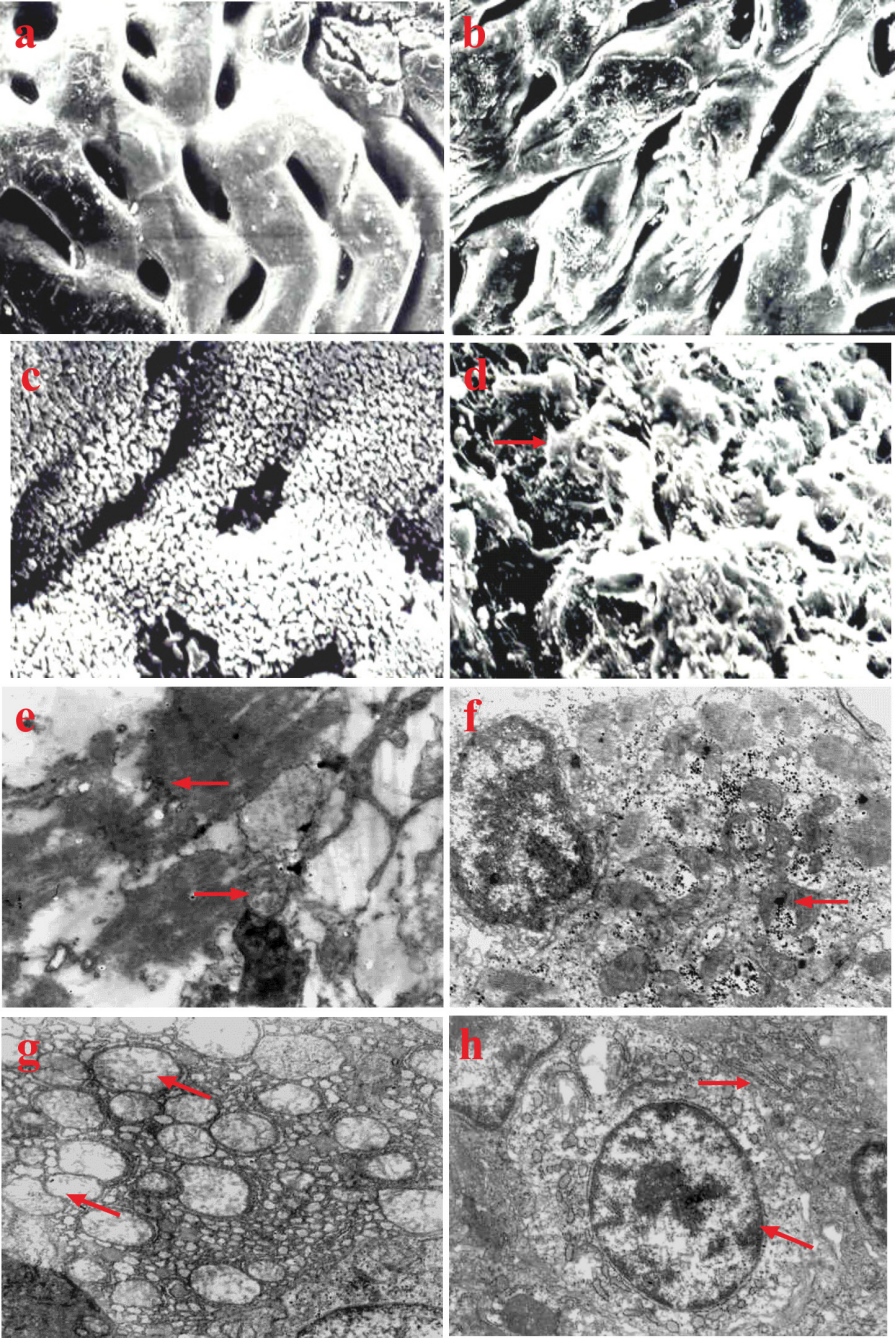
Ultrastructural changes in vitamin E deficient fish **a**. Intestinal mucosa of normal fish in the control group, × 5000. **b**. Intestinal mucosa epithelium falls off in vitamin E deficient fish, × 5000. **c**. Microvilli of intestinal mucosa epithelium of normal fish in the control group, × 5000. **d**. Microvilli of intestinal mucosa epithelium fall off in vitamin E deficient fish (→), × 5000. **e**. Structure of skeletal muscle becomes obscure with a dissolving sarcoplasm (←). Mitochondria swelling with crista disintegration (→), × 20000. **f**. Myocardium mitochondria become swollen and obscure with the appearance of glycogen granule inclusions inside (←), × 12,000. **g**. Mitochondria of liver cells swelling with the cristas lysis (↖), the rough endoplasmic reticulum distending with ribosomes falling off, × 10,000. **h**. Swelling and increased volume of the mitochondrion of renal tubular epithelial cell is observed (↖). The rough endoplasmic reticulum is distended with the desquamation of ribosome particles (→), × 10,000.

By the 10^th^ week, the intestinal mucosa epithelium of fish in group I had fallen off (Figure 3b). The microvilli of epithelium also fell off, exposing rough lamina propria in some areas (Figure 3d). Similar lesions occurred successively in groups II and III fish after the 12^th^ week, while there were no obvious changes in group IV fish (Figure 3a, 3c).

#### Skeletal muscles

The skeletal muscle structure of fish in group I became obscure alongside dissolving sarcoplasm and swollen mitochondria with crista disintegration at the 12^th^ week (Figure 3e). Other changes including sarcoplasm solidification and nuclear pyknosis could be observed in the most severely affected areas. Fish in groups II and III presented similar changes successively by the 14^th^ week.

#### Cardiac muscles

At the 10^th^ week, the myocardium mitochondria of fish in group I became swollen and obscure, and enlarged in volume, along with the presence of some glycogen particles in the mitochondria (Figure 3f). Similar changes appeared in groups II and III fish after the 12^th^ and 14^th^ week, respectively.

#### Liver

The mitochondria of liver cells in group I fish became swollen and enlarged in volume, with the cristas breaking and falling into the cystic cavity (Figure 3g). In addition, the rough endoplasmic reticulum distended and ribosomal particles had fallen off by the 10^th^ week. Fish in groups II and III presented similar changes after the 12^th^ and 14^th^ week, respectively.

#### Kidney

By the 10^th^ week, the mitochondria in renal tubular epithelial cells were swollen with an enlarged volume in group I fish. The rough endoplasmic reticulum became distended with ribosomes falling off, and the mitochondrion of glomerular podocyte became swollen with blurring crista (Figure 3h). Similar changes were observed in fish from groups II and III at the 12^th^ and 14^th^ week, respectively.

### Changes in blood and serum oxidative parameters

**Figure 4:**
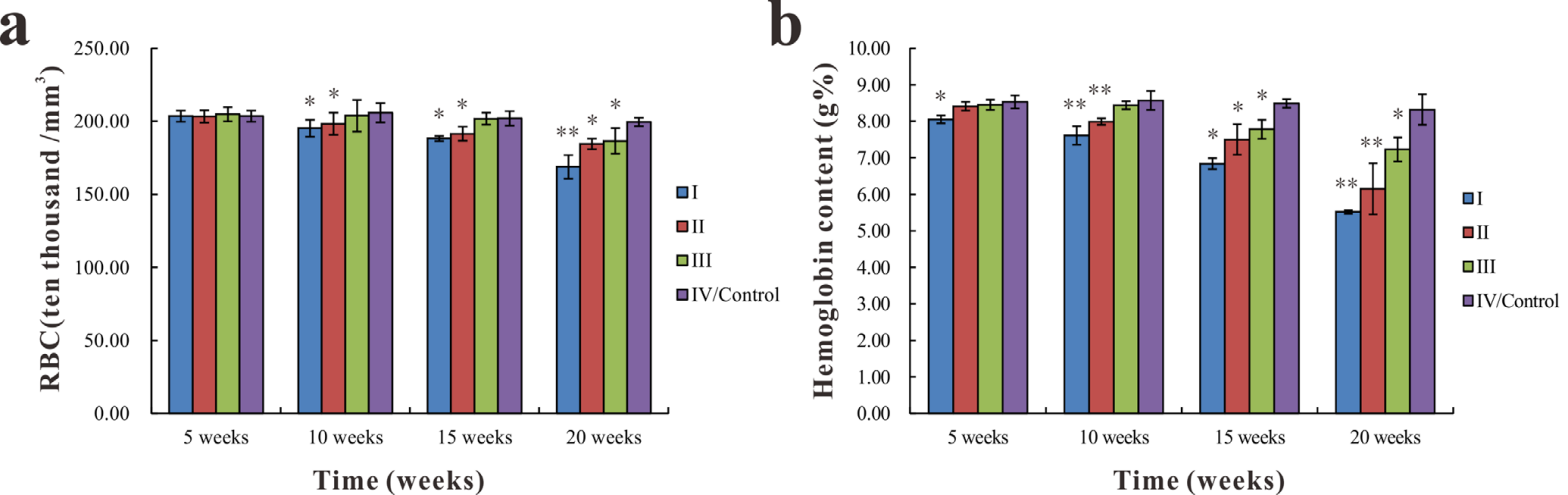
Changes in RBC count and hemoglobin content in common carp among the different groups **a**.. Changes in RBC count **b**.. Changes in hemoglobin content. Data presented as the mean ± standard deviation (*n* = 5); * indicates significant difference compared with the control group (*p* < 0.05); ** indicates very significant difference compared with the control group (*p* < 0.01).

As shown in Figure 4a, there were no significant differences in RBC count among groups at the 5^th^ week. While the RBC count was significantly lower (*p* < 0.05 or *p* < 0.01) in groups I and II than in group IV at the 10^th^, 15^th^, and 20^th^ week, it was significantly lower in group III (*p* < 0.05) compared with group IV at the 20^th^ week. The hemoglobin content in group I was significantly lower (*p* < 0.05) than group IV at the 5^th^ week. At the 10^th^, 15^th^, and 20^th^ week, hemoglobin content was significantly lower (*p* < 0.05 or *p* < 0.01) in groups I and II than in the group IV. The hemoglobin content of group III was significantly lower (*p* < 0.05) than in group IV at the 15^th^ and 20^th^ week (Figure 4b).

**Figure 5:**
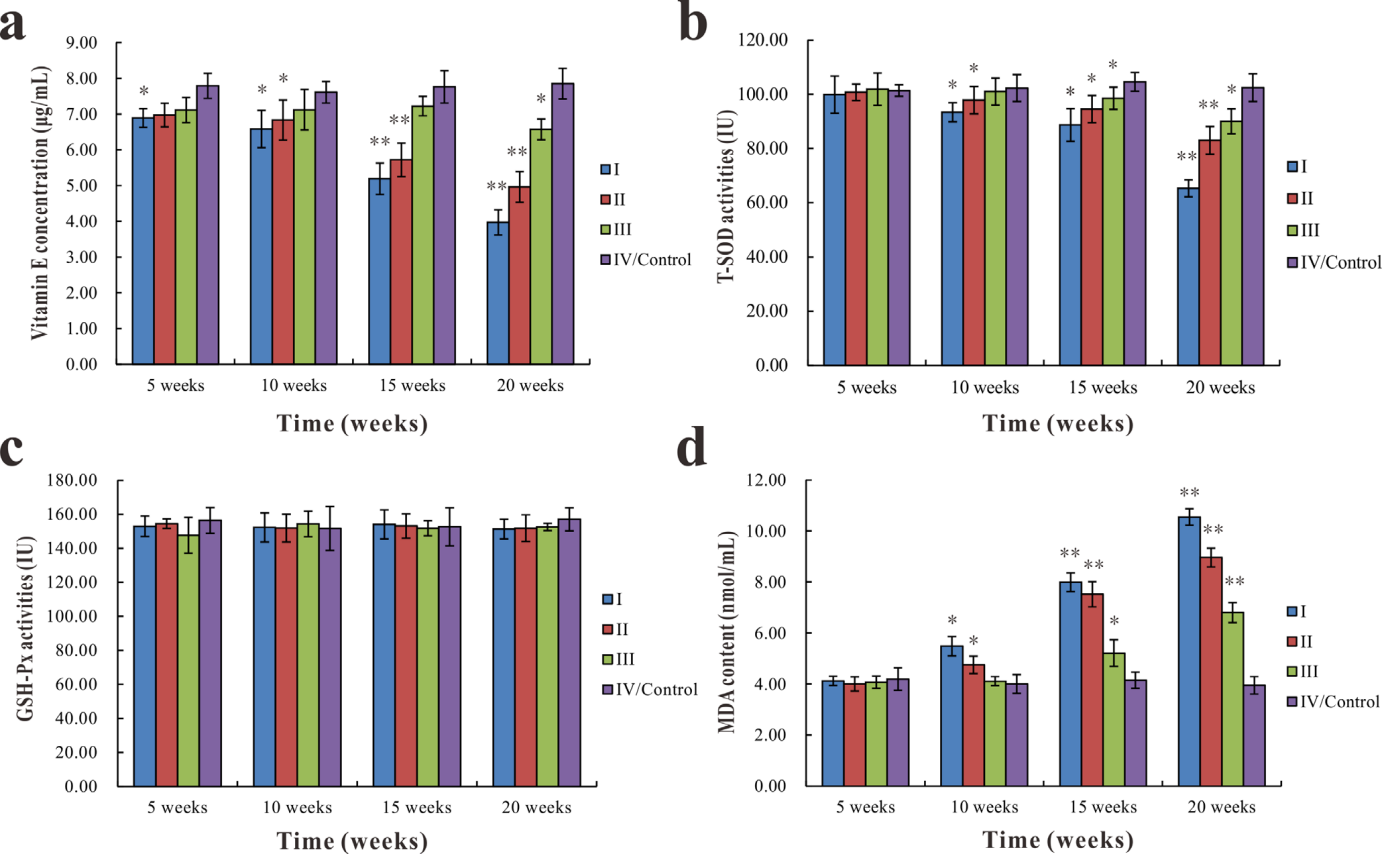
Changes in vitamin E concentration, T-SOD and GSH-Px activities, and MDA content in the serum of common carp among different groups Changes in vitamin E concentration **a**., T-SOD activities **b**., GSH-Px activities **c**., and MDA serum content **d**.. Data presented as the mean ± standard deviation (*n* = 5); * indicates significant difference compared with the control group (*p* < 0.05); ** indicates very significant difference compared with the control group (*p* < 0.01).

The serum vitamin E concentration of group I was significantly lower (*p* < 0.05) than group IV at the 5^th^ week. At the 10^th^, 15^th^, and 20^th^ week, serum vitamin E concentration significantly decreased (*p* < 0.05 or *p* < 0.01) in groups I and II compared with group IV. The serum vitamin E concentration in group III was significantly lower (*p* < 0.05) than in group IV only at the 20^th^ week (Figure 5a). There were no significant differences in T-SOD activities and MDA content among different groups at the 5^th^ week. The T-SOD activities of groups I, II, and III were significantly lower (*p* < 0.05 or *p* < 0.01) than in group IV at the 10^th^, 15^th^, and 20^th^ week, except group III at the 10^th^ week (Figure 5b). In contrast, the MDA content of groups I, II, and III markedly increased (*p* < 0.05 or *p* < 0.01) compared with group IV at the same time (Figure 5d). However, there were no significant differences in GSH-Px activities among groups throughout the entire experiment (Figure 5c).

## DISCUSSION

Common carp have a higher dietary vitamin E requirement compared with many other fish species; the National Research Council recommends a supplementation of 100 IUkg^−1^ [13]. In this study, common carp were fed feedstuff with different vitamin E supplementations (0, 25, 50, and 100 IUkg^−1^) for 20 weeks to investigate the effects of dietary vitamin E deficiency on pathological changes and serum oxidative stress. The results showed a negative correlation between the morbidity and mortality of the experimental groups and dietary vitamin E supplementation; the lower the dietary vitamin E supplementation, the higher the morbidity and mortality. It has been reported that vitamin E deficiency in livestock and poultry can cause white muscle disease, nutritional liver disease, exudative diathesis, pancreatic atrophy, and genetopathy [4, 14]. Similarly, fish also exhibit pathological lesions when deficient in vitamin E. In the present study, vitamin E deficiency in common carp resulted in anemia, malformation (rachiocamposis and tail upwarping), exudative diathesis (muscle edema, exophthalmia, leprnorthsis, and ascites), sekoke disease, and nutritional liver disease. These findings constitute a basic pathological model of common carp with vitamin E deficiency, and are comparable to the pathological changes observed in vitamin E deficient rainbow trout [10], rockfish [11], tilapia [12], and grass carp [2].

In the current study, sekoke disease was a prominent symptom of vitamin E deficiency in common carp. Morphological changes comprised a thin back (back muscle thickness was 1/4-1/2 that of the control group) and sunken back muscles on both sides causing presentation of a blade-shaped back. Histopathological changes of sekoke disease presented nutritional myopathy characterized by muscle fiber denaturation and necrosis (with dissolving and disappearing fibers in some cases), and attenuation of the residual muscle fibers exhibiting atrophy, similar to the pathological changes of skeletal muscles induced by vitamin E deficiency in pigs, rabbits, dogs, and cats [15-19].

Exudative diathesis and fish body malformation were two other important changes observed in this study. Sick fish presented muscle edema, exophthalmia, leprnorthsis and ascites, rachiocamposis, and tail upwarping, consistent with the pathological changes of grass carp [2] and rockfish [11] with vitamin E deficiency. The occurrence of exudative diathesis may be due to the fact that vitamin E deficiency led to lipid peroxidation and vascular endothelial cell injury, breaking the link between endothelium cells, even resulting in degeneration and necrosis. This then caused an increase in vascular wall permeability, with plasma extravasation and edema. The accumulation of edema fluid in the scale follicle, at the bottom of the eyeballs, and in the abdominal cavity caused leprnorthsis, exophthalmia, and ascites, respectively.

When common carp lacked dietary vitamin E, the pathological lesions of the liver mainly presented hepatocyte vacuolar degeneration, presence of difference-sized empty vesicles in cells, occurrence of necrolysis forming dissolving stoves, and numerous round or oval ceroid pigment depositions in the liver cells. Vitamin E has an antioxidant capacity, which blocks the peroxidation of unsaturated fatty acid into ceroids. In the vitamin E deficient fish in the present study, peroxidation of unsaturated fatty acid occurred resulting in ceroid pigment deposition. In addition, vascular endothelial cells of the interlobular vein became impaired, the vascular wall permeability increased, and plasma extravasation occurred, resulting in vascular wall fibrinoid degeneration and the formation of mixed thrombus in the lumen. This was consistent with the pathological changes observed in vitamin E deficient pigs [16, 17].

In addition, pancreatic pathological lesions in vitamin E deficient fish mainly exhibited glandular cell denaturation, which dissolved and disappeared, in accordance with the pathological changes observed in vitamin E deficient chicken [20]. Insulin excreted by the endocrine portion of the pancreas can catalyze glucose oxidation reaction, glycogenesis, lipogenesis, and adenosine triphosphate engendering. It also promotes myoprotein synthesis, inhibits protein decomposition, and stimulates skeletal muscle nutrition metabolism [21, 22]. Therefore, severe pancreatic lesions would inevitably cause a reduction in insulin secretion, skeletal muscle malnutrition, protein synthesis inhibition, and increase protein decomposition. This presents as muscle fiber atrophy and morphological diminution. Furthermore, compared with the control group, the RBC count, hemoglobin content, and vitamin E concentration significantly decreased in vitamin E deficient fish, indicating that vitamin E deficiency can cause anemia in common carp, in agreement with the findings in rainbow trout [10]. In vitamin E deficient fish, unsaturated fatty acids of red cell membranes were attacked by free radicals, which promoted lipid peroxidation and caused membrane damage, resulting in increased cell brittleness and hemolytic anemia.

Serum MDA content, a major index of lipid peroxidation *in vivo*, was considerably higher in vitamin E deficient fish than in control fish. This altered membrane fluidity and increased membrane fragility leading to aggravating lipid peroxidation and oxidative damage of the cellular biomembrane structure [23-25]. This was also shown by degeneration and necrosis of tissues and cells in the heart, liver, spleen, and skeletal muscles in the histopathological analysis. SOD and GSH-Px, as endogenous antioxidants, have the capability to prevent the uncontrolled formation of reactive oxygen negative ions and resist against oxidative damage [26]. In the present study, the lower the dietary vitamin E supplementation, the lower the serum SOD activities, which led to an excessive accumulation of superoxide and hydrogen peroxide and in turn generated hydroxyl radicals involved in the initiation and propagation of lipid peroxidation [27]. In contrast, GSH-Px levels were unaffected in vitamin E deficient fish compared with control fish, consistent with the findings in rats [28, 29].

## MATERIALS AND METHODS

### Fish and diets

A total of 320 healthy common carps (*C. carpio*; average weight 60 ± 10 g) were purchased from a commercial fish farm in Chengdu, Sichuan Province, China. The fish were domesticated in tanks (80 × 60 × 30 cm^3^) for a week before the experiment. The tanks were continuously oxygenated. During the experiment, water temperature was maintained at 23-26°C, with a pH of 6.8-7.3 and dissolved oxygen of 8-10 mg/L. Fish were fed with basal diet at the rate 3% of fish body weight three times a day. The basal diet was prepared according to Table 3 [30, 31].

**Table 3:** The composition and nutrient level of basal diet of common carp

Ingredients	Content (%)			
	I	II	III	IV
Casein	38.00	32.00	32.00	32.00
Gelatin	9.00	8.00	8.00	8.00
Dextrin	28.00	28.00	28.00	28.00
Cellulose acetate	14.00	18.00	18.00	18.00
Carboxymethyl cellulose	2.00	2.00	2.00	2.00
Fish oil	3.00	6.00	6.00	6.00
Soybean oil	3.30	3.30	3.30	3.30
Calcium phosphate	0.90	0.90	0.90	0.90
Potassium phosphate	0.92	0.92	0.92	0.92
Salt	0.20	0.20	0.20	0.20
Mineral premix ^a^	0.50	4.00	4.00	4.00
Vitamin premix ^b^	0.08	0.08	0.08	0.08
Choline chloride	0.10	0.10	0.10	0.10
Total	100	100	100	100
Nutrient level (%)				
Crude protein	39.30	36.34	36.34	36.34
Crude fat	6.30	6.30	6.30	6.30
Lysine	2.88	2.88	2.88	2.88
Met+ Cys	1.20	1.20	1.20	1.20
Calcium *	0.68	0.68	0.68	0.68
Available phosphorus *	0.73	0.73	0.73	0.73

a Mineral element addition level (mgkg^-1^): Fe 150, Zn 50.00, Mn 20.00, Cu 3.00, I 0.50.

b Vitamin addition level (mgkg^-1^): V_A_ 8000 IU, V_D_ 2000 IU, V_K_ 3.00, V_C_ 100.00, V_B1_ 5.00, V_B2_ 8.00, V_B6_ 6.00, V_B12_ 12.00, calcium pantothenate 30.00, niacin 40.00, biotin 1.00, folic acid 1.00, myoinositol 80.00

### Grouping trial

Fish were equally divided into two treatments (160 fish / treatment); one treatment was used for sampling and dissecting, the other one was used for the observation and statistical analysis of fish performance during the whole experiment. Fish in each treatment were randomized into four groups (40 fish/ group) and fed purified diets containing vitamin E at the following concentrations 0 IUkg^−1^ (group I), 25 IUkg^−1^ (group II), 50 IUkg^−1^ (group III), or 100 IUkg^−1^ (group IV; the control group, fed the standard cyprinoid feedstuff vitamin E supplementation [32] ). Fish were anesthetized with MS222 prior to dissecting and blood was sampled from the caudal vein.

The entire experiment lasted for 20 weeks, and the experiments involving the use of fish and all experimental procedures were approved by Experimental Animal Operating Norms and Welfare Management Committee, Sichuan Agricultural University.

### Clinical symptoms and histopathological changes

Fish performances were observed daily, and clinical symptoms including sekoke disease (i.e., thin-backed), which was characterized by back muscle atrophy, exophthalmia, leprnorthsis, and rachiocamposis, were recorded carefully. Two fish in each group were humanely euthanized using MS222 anesthetic. The skin, muscles, eyes, gills, brain, heart, liver, pancreas, spleen, head kidney, hind kidney, and gonads were removed, fixed in Bouin's Fluid and dehydrated in ethanol, followed by paraffin sectioning at 5 μm, and hematoxylin-eosin staining. Histopathological changes were observed under a microscope (Nikon, Tokyo, Japan).

### Ultrastructural observations

Intestines of the sacrificed fish with obvious symptoms were fixed with 2.5% glutaric dialdehyde, dehydrated in acetone, followed by drying and coating at critical point, then observed with a scanning electron microscope (SEM, JEOL, Japan). At the same time, the heart, liver, skeletal muscle, and kidneys were also excised from the fish with obvious symptoms and fixed with 2.5% glutaric dialdehyde, dehydrated in ethanol, and embedded in epoxy resin for ultrathin sectioning. The sections were stained with uranyl acetate and lead citrate, and observed under transmission electron microscope (TEM, JEOL, Japan).

### Sample preparation

Five fish from each group were phlebotomized from the caudal vein at the 5^th^, 10^th^, 15^th^, and 20^th^ week during the experiment. The blood was divided into two groups, one group with added heparin sodium anticoagulation was used to determine the RBC count and hemoglobin content, the other one was centrifugated for 20 minutes at 4000 r/min at 4 °C to collect the serum for measurements of vitamin E concentration, SOD and GSH-Px activities, and MDA content.

### Detection of oxidative stress parameters in the serum

The RBC count was determined *via* the microscope count method and hemoglobin content was assessed by spectrophotometry according to the physiology experimental course [13]. The vitamin E concentration, SOD and GSH-Px activities, and MDA serum content were detected using biochemical methods following the instructions of reagent kits purchased from Nanjing Jiancheng Bioengineering Institute of China (vitamin E: Cat.No.A008; SOD: Cat.No.A001-1; GSH-Px: Cat.No.A005; and MDA: Cat.No.A003-1). The absorbance of vitamin E, SOD, GSH-Px, and MDA were measured at 533 nm, 550 nm, 412 nm, and 532 nm, respectively using a microtiter plate reader (Thermo, Varioskan Flash, USA).

### Statistical analysis

The significance of differences among the four groups were analyzed by analysis of variance using SPSS 19.0 (SPSS, Chicago, IL, USA), and data was presented as mean ± standard deviation. A value of *p* < 0.05 was considered significantly different and *p* < 0.01 was considered very significantly different.
